# A cross-sectional study assessing barriers and facilitators to the sustainability of physical activity and nutrition interventions in early childhood education and care settings

**DOI:** 10.1186/s12966-024-01699-z

**Published:** 2025-01-03

**Authors:** Noor Imad, Alix Hall, Nicole Nathan, Adam Shoesmith, Nicole Pearson, Melanie Lum, Alice Grady, Erin Nolan, Serene Yoong

**Affiliations:** 1 Global Centre for Preventive Health and Nutrition, Institute for Health Transformation, School of Health and Social Development, Faculty of Health, Deakin University, Burwood, VIC 3125 Australia; 2 Hunter New England Population Health, Wallsend, NSW 2287 Australia; 3 School of Health Sciences, Department of Nursing and Allied Health, Swinburne University of Technology, Hawthorn, VIC 3122 Australia; 4 School of Medicine and Public Health, College of Health, Medicine and Wellbeing, University of Newcastle, Callaghan, NSW 2308 Australia; 5 Hunter Medical Research Institute, New Lambton Heights, NSW 2305 Australia; 6 The National Centre of Implementation Science (NCOIS), The University of Newcastle, Newcastle, NSW Australia

**Keywords:** Children, Early childhood education and care, Physical activity, Nutrition, Prevention barriers, Facilitators, Sustainment, Sustainability

## Abstract

**Background:**

Effective evidence-based physical activity and nutrition interventions to prevent overweight and obesity and support healthy child development need to be sustained within Early Childhood Education and Care (ECEC) services. Despite this, little is known about factors that influence sustainability of these programs in ECEC settings. Therefore, the aim of this study was to describe the factors related to sustainability of physical activity and nutrition interventions in ECEC settings and examine their association with ECEC service characteristics.

**Methods:**

A cross-sectional study was undertaken with a nationally representative sample of 473 Australian ECEC services. Factors related to the sustainability of ECEC-based physical activity and nutrition interventions were assessed using the validated Integrated Measure of PRogram Element SuStainability in Childcare Settings (IMPRESS-C), measuring Outer Contextual Factors, Inner Contextual Factors, Processes and Characteristics of the Intervention domains for interventions that supervisors reported as currently implementing. Participants responded using a 5-point Likert scale, with responses ranging from 1 (completely disagree) to 5 (completely agree). Domain scores were calculated for each service by averaging item responses. Linear regression models between ECEC service characteristics and the IMPRESS-C domains were undertaken.

**Results:**

Data from 473 Australian childcare services nationally found that the domains: Processes ($\overline{x }$=3.78, SD = 0.64), consisting of partnership/engagement and training/support/supervision; and Outer Contextual Factors ($\overline{x }$=3.93, SD = 0.63), including policy and legislation, and socio-political context had the lowest mean scores indicating they may likely be barriers to sustainability. Linear regression analyses revealed no statistically significant associations between examined factors and ECEC service characteristics. There was a statistically significant association between the number of years services delivered their interventions and the Characteristics of the Intervention domain (*p* = 0.035) suggesting that this domain may influence sustainability of programs.

**Conclusions:**

This study suggests that factors related to the Processes and Outer Contextual Factors domains had the lowest scores and as such, strategies to support the sustainability of physical activity and nutrition interventions implemented in ECEC settings may need to consider how to best address these factors.

## Background

Overweight and obesity are major risk factors and are associated with leading causes of poor health and early death, contributing to significant health and economic challenges globally [1]. In 2020, 39 million children under five years worldwide were classified as overweight or obese [2]. Low levels of physical activity and suboptimal diets are primary risk factors for excess body weight [3]. The World Health Organization (WHO) recommends supporting the development of healthy behaviours such as physical activity and healthy eating in children at a young age as a way of preventing excess weight gain and improving child wellbeing more generally, given habits developed early in life track into adulthood [4–8]. Promoting healthy eating and physical activity in children is essential for their growth and development, as it helps prevent obesity, supports mental and physical health, and reduces future risks of chronic illnesses such as diabetes and heart disease [9, 10].

Key international and national bodies have recommended Early Childhood Education and Care (ECEC) services (including preschools, family day care, long day care, kindergarten and nurseries) as an ideal setting to reach young children with obesity prevention efforts as they provide care to 87% of children aged 3–5 years globally [9, 11–13]. In addition, children aged 3–5 years spend an average of 25–35 h a week in ECEC services, and therefore, are an important environment to influence behaviour change [14]. In recent years there has been growth in the empirical evidence demonstrating the positive impact of ECEC-based physical activity and healthy eating interventions on children’s health [15–20]. Findings from systematic reviews suggest that interventions that include opportunities for adult-led, structured child activity [21], staff encouraging physical activity [22], opportunities for children to develop gross motor and movement skills [23], parents packing of lunchboxes [24], increasing access to fruit and/or vegetables [25], and healthy eating educational activities [26] can improve child physical activity, dietary behaviours and/or obesity outcomes. In order to realise the population health impact of these programs, it is important they are both implemented sustained long-term.

Sustainability is defined as the ongoing delivery of a program “(1) measured after a defined period of time, (2) the program, clinical intervention, and/or implementation strategies continue to be delivered and/or (3) individual behaviour change (i.e., clinician, patient) is maintained; (4) the program and individual behaviour change may evolve or adapt while (5) continuing to produce benefits for individuals/systems” [27]. Ensuring public health interventions are sustained, is important to: (i) realise the public health impact of such programs; (ii) protect the significant resource allocation and public health investment in program delivery in this setting; and (iii) foster community trust and confidence in the delivery of future programs [28–30].

Despite the importance of sustainability, evidence suggests that up to 40% of all new public health interventions, are not sustained beyond the first few years after termination of initial implementation support [31–33]. Similarly, studies in ECEC settings, schools and more broadly examining public health initiatives indicate that implementation is most likely to attenuate after support is withdrawn [28, 33–36]. To help address this, it is important to develop a comprehensive understanding of the factors that influence intervention sustainability [28, 37, 38]. Employing theoretical frameworks such as the Integrated Sustainability Framework [38], can provide a holistic approach to understanding the potential factors influencing sustainability of these interventions. The Integrated Sustainability Framework helps to identify and organise multi-level factors important in facilitating sustainability, informed by available empirical research [38, 39]. This allows for a comprehensive assessment and addressing of these determinants. A number of validated measures exist to do this, such as the Program Sustainability Assessment Tool [40] or the Integrated Measure of PRogram Element SuStainability in Childcare Settings (IMPRESS-C) [41], the latter of which was developed by the research team to assess constructs related to sustainability, specifically in ECEC settings. Using such frameworks and measures to assess the factors influencing sustainability is crucial for guiding the development of strategies that address and overcome experienced barriers, ultimately supporting intervention sustainment. Sustainment is defined as “the sustained use or delivery of an intervention in practice following external implementation support” [39, 42, 43].

There are, however, a lack of studies examining sustainability of ECEC-based physical activity and healthy eating interventions. A review by Shoesmith and colleagues (2021) identified that only two of the 31 included articles (6%) explored the barriers of sustaining evidence-based interventions (EBIs) in ECEC settings [39]. Consistent with this review, Asada and colleagues (2022) found that only six of 24 studies (25%) (of which two of these studies were included in the review by Shoesmith and colleagues (2021)) reported findings related to the barriers and facilitators to sustainability of physical activity and healthy eating interventions in ECEC settings with children aged 2–5 years [44]. Collectively, these reviews highlighted the most common reported on factors influencing sustainability to be: (1) integration of the program within the existing curricula, (2) available resources, such as high staff turnover, staff motivation, parent engagement, resistance to change and available equipment, (3) financial resources, cost effectiveness, and, (4) staff trainings, executive/leadership support [39, 44]. Together, these reviews identified only six studies focusing on the factors of sustainability in ECEC settings [39, 44]. A large proportion of these studies were conducted in school settings or involved small sample sizes ranging from four to 113 educators in ECEC services [39, 44]. Further, these studies may have overlooked important determinants influencing the sustainability of EBIs, given the lack of application of theoretical frameworks to explore barriers and facilitators as well as the lack of validated measures to identify such determinants in the ECEC studies included in these reviews.

Additionally, there is a limited exploration of barriers and facilitators of program sustainability by different service characteristics such as low-resource settings [45]. Factors such as socio-economic status, rurality, service type, size, and operating hours are associated with implementation and have been theorised to impact on an EBI’s sustainability in several studies but have not been examined in regards to sustainability [46, 47]. Therefore, exploration of barriers by such service characteristics are needed to better understand potential differences by these socio-demographic characteristics.

To address this gap, this study aimed to describe the barriers and facilitators related to the sustainability of physical activity and healthy eating interventions in ECEC services using a validated measure of sustainability determinants; IMPRESS-C [41]. In addition, this study aims to explore the association between the barriers and facilitators to the sustainability of physical activity and healthy eating interventions in ECEC settings and various ECEC service characteristics including socio-economic status, rurality, service type, size, and operating hours.

## Methods

### Study design and setting

A cross-sectional study was undertaken with a random sample of Australian ECEC services currently implementing selected physical activity or healthy eating interventions between August 2021 and May 2022. ECEC services included long day care and preschools, in which long day care services usually operate from 7am to 6 pm Monday through Friday and cater for children from birth to school age, while preschools cater for children from 3–5 years and usually operate from 9am to 3.30 pm [48]. Services were eligible if they were a centre-based ECEC service (long day care and preschools) approved by the Australian Children Education and Care Quality Authority (ACECQA), which provides guidance, resources and services to support the sector to improve outcomes for children [49]. ECEC services were not eligible if they were: (1) a family day care service or an outside school hours care service; (2) a Department of Education primary or central school due to differing ethical requirements; (3) closed (4) catered exclusively to children requiring specialist care; (5) did not have a staff member with sufficient English to complete the survey; or (6) were a service located in the Hunter New England Local Health District (HNELHD) as they were participating in other surveys to assess implementation of physical activity and healthy eating research. Ethical approval was provided by Hunter New England Human Research Ethics Committee (HREC) (06/07/26/4.04 2019/ETH12353) and ratified with the University of Newcastle HREC (H-2008–0343) and Deakin University HREC (2023–062).

### Study recruitment and procedures

Across Australia, 2,050 ECEC services were randomly selected from the ACECQA national register and were invited to participate in an online or telephone survey for the broader study [50]. Both online and telephone approaches were utilised to maximise survey completion rate consistent with previously undertaken processes by the team. Services were recruited using a staggered approach, whereby services were invited via email and mail to participate in the online survey. Each email contained a link that directed them to an information statement and the online survey. A reminder email was sent approximately one week after the initial invitation. If the survey was not completed online within one week, services were called by trained interviewers and invited to complete the survey over the phone. The nominated supervisor, or another staff member with knowledge of the service implementation of physical activity and healthy eating programs, responded to items on behalf of the service.

### Data collection procedures and measures

Data collection occurred between August 2021 and May 2022. Services were assessed on 13 selected physical activity or healthy eating interventions determined as evidence-based via systematic review evidence [20, 51]. The specific interventions are outlined in Appendix 1 and are consistent with that recommended by national and international policies to improve child health and wellbeing. To reduce participant burden, allocation of this survey was based on the services current implementation status of these interventions (i.e. implementing based on set criteria) and a predetermined hierarchy to improve the likelihood of equal distribution. While no active program was delivered by the research team to support implementation, there are a number of state-wide programs in Australia (e.g. Munch &Move, the Achievement Program) that work with ECECs to implement healthy eating and physical activity interventions.

#### ECEC service characteristics

Service demographic information was collected during the online or telephone interview with the service and included type of centre (i.e., long day care or preschool), number of full-time, part-time, and casual educators working at the service, and number of children enrolled. The role of the responder was also captured. Service postcode was obtained from the ACECQA national register and Australian ECEC service postcodes ranked in the top 50% according to the 2016 Socio-Economic Indexes for Areas (SEIFA) were classified as least disadvantaged (i.e., high socio-economic status), whilst the lower 50% of postcodes were classified as most disadvantaged (i.e., low socio-economic status) [52]. The Australian Statistical Geography Standard was used to classify service locality as either urban or regional/remote [53].

#### Barriers and facilitators associated with the sustainability of physical activity and healthy eating interventions in ECEC services

Online or telephone surveys were used to assess the barriers and facilitators to the sustainability of physical activity and healthy eating interventions. The interventions assessed in the current study were based on extensive reviews indicating likely positive outcomes on child behaviours [54] – see Appendix 1 for definitions. These selected interventions included; four physical activity interventions; activities with children (defined as engaging children with activities at least once per week), Energisers (defined as three, five minute educator led activity breaks [55] on more than one day per week), providing fundamental movement skills (defined as an activity to intentionally develop fundamental movement skills at least one day a week) and outdoor time with a planned activity at least once per week, and nine healthy eating interventions; two or more serves of fruit per day, two or more serves of vegetables per day, exposure to different vegetables at least once per month, healthy eating-themed special days at least once per month, interactive healthy eating activities at least once per month, observations of children’s lunchboxes at least one to two times per week, planned healthy eating lessons at least monthly, play based healthy eating activities at least once per month, and strategies to encourage consumption of age appropriate beverages at least two times per week (see Appendix 1). Barriers and facilitators were identified using the IMPRESS-C [41]. This measure was developed based on the Integrated Sustainability Framework [38], a framework informed by available empirical research on factors identified as important determinants of sustainability across a range of contexts and interventions. The 26-item IMPRESS-C examines sustainability determinants across four domains including: Outer Contextual Factors (3 items) e.g., the socio-political context or the funding environment; Inner Contextual Factors (8 items) e.g., financial resources, program champions, and organisational support; Processes (5 items) e.g., training, stakeholder engagement and partnerships; and Characteristics of the Intervention (10 items) e.g., adaptability, fit within the context and population [41] (see Appendix 2). The IMPRESS-C was developed and validated by the research team for completion of service executives within the ECEC setting (details published elsewhere) [41], which was then used in the current study. In a sample of 405 ECEC services, the IMPRESS-C displayed good structural validity (Standardized Root Square Residual = 0.056, Comparative Fit Index = 0.993, Root Mean Square Error of Approximation = 0.067), and illustrated: good internal consistency (Cronbach’s *a*: 0.53 to 0.92); emerging concurrent validity; good norms, and good overall pragmatic qualities (cost, readability, length, and assessor burden) [41]. An overview of the measure is provided in Table 1. Service nominated supervisors were asked to rate their level of agreement to items based on a 5-point Likert scale from ‘1’ (completely disagree) to ‘5’ (completely agree).

**Table 1:** Overview of the IMPRESS-C domains [41]

IMPRESS-C Measure Domain	Context/Description of factors covered	Number of items	Example item
Outer contextual factors	• Policy and legislation • Funding environment • External leadership • Values, needs, and priorities • Sociopolitical context	3	*“My service governing body has a policy or guideline regarding the ongoing delivery of ****“the program”***^***a***^* that my service follows. (Note: A governing body refers to an educational department or authority e.g., Australian Children's Education & Care Quality Authority).”.*
Inner contextual factors	• Service Champions • Organisational resources/funding • Staffing/turnover • Structural characteristics	8	*“My service would be able to continue to deliver ****“the program”***^***a***^* if there was a change of leaders (e.g., management or champions) at our service.”.*
Processes	• Training/support/supervision • Program evaluation/data • Technical support • Partnership/engagement	5	*“My service promotes the ongoing delivery of ****“the program”***^***a***^* to the wider service community e.g., through a website or newsletter. (Note: service community refers to administrators, teachers/educators, staff members, children, their parents/guardians and families directly involved with your service).”.*
Characteristics of the intervention	• Perceived benefit/need • Adaptability • Burden/complexity • Cost	10	*“My service is able to adapt ****“the program”***^***a***^* if resources/equipment are reduced.*

^a^ “the program” refers to the intervention that the ECEC service is currently implementing

### Analysis

Data was analysed in R 4.0.3 [56]. Descriptive statistics, including median, interquartile range (IQR), minimum, maximum, means, and standard deviations, were calculated for each of the four domains; Outer Contextual Factors, Processes, Inner Contextual Factors, Characteristics of the Intervention domains. These descriptive statistics were also used to describe ECEC service characteristics and provide mean score for each domain for physical activity and healthy eating interventions overall and for each individual intervention. The frequency (percentage) of responses for each survey question is presented. Mean values were used to describe domains as potential barriers and facilitators [57]. No cut points for classifying barriers were selected however lower domains scores were considered as more likely to be barriers, while higher domain scores were considered as more likely to be facilitators. To investigate the association between each ECEC service characteristic and domain score, linear regression models were also run between service socio-economic status (as classified using service postcode), rurality, service type, service size, operating days and hours, and overall mean score for each IMPRESS-C domains; Outer Contextual Factors, Processes, Inner Contextual Factors, Characteristics of the Intervention domains (i.e., barriers and facilitators perceived to influencing sustainability of physical activity and healthy eating interventions). The domain scores were modelled individually as fixed effects. Socio-economic status, Accessibility/Remoteness Index of Australia, service type and service size were dichotomised. For socio-economic status, the bottom five Australian Bureau of Statistics deciles were considered lower socio-economic status, while the top five deciles were considered higher socio-economic status [58]. A binomial distribution with a logistic link was used for socio-economic status, Accessibility/Remoteness Index of Australia, service type and service size, while a normal distribution with an identity link was used for number of days open and hours of operation. The association between whether the service had delivered their health promotion program for ≥ 2 years, and the domain score was modelled using a generalised linear model with a binomial distribution and logistic link. The odds ratio with corresponding 95% confidence intervals and p-values were presented. The reference group was “delivered program < 2 years”. Additionally, any statistically significant differences in service socio-economic status area/geographical location between consenters and non-consenters were examined. Statistical significance was defined as *p* < 0.05. “Refused” and “Don’t know” responses for the survey were imputed using a single imputation with predictive mean matching (0 knots). If a participant was “Refused” / “Don’t know” for all responses to the survey, then they were excluded. The imputed data were included for all analyses.

## Results

Of the 2,050 ECEC services that were invited to participate in the larger study, 993 ECEC services completed the survey. Following contact, ECEC services consented to the study and were assessed for eligibility, with 116 (6%) services deemed ineligible. This was most commonly due to the services being part of a Department of Education primary or central school. Overall, 473 ECEC services responded to the sustainability items based on the intervention (either physical activity or healthy eating) that they were implementing.

The demographic characteristics of services which received items around sustainability are described in Table 2. The majority of the services were long day care services (*n* = 430, 91%). Approximately 59% (*n* = 281) of ECEC services were in high socioeconomic area and 93% (*n* = 442) were located in a major city (see Table 2).

**Table 2:** Demographic characteristics of participating ECEC services

Service (*n* = 473)	*n*	%
***Type of service:***		
Long day care	430	90.9%
Preschool	43	9.1%
***Position:***		
Director	155	32.8%
Nominated supervisor	255	53.9%
Other^a^	63	13.3%
***Socio-Economic Indexes for Areas (SEIFA)***		
Most disadvantaged (low socio-economic status)	192	40.6%
Least disadvantaged (high socio-economic status)	281	59.4%
***Geographic Location:***		
Urban (major cities)	442	93.4%
Regional/remote (inner regional, outer regional, remote)	31	6.6%
***Service State***		
Australian Capital Territory	11	2.3%
New South Wales	199	42.1%
Queensland	95	20.1%
South Australia	20	4.2%
Tasmania	11	2.3%
Victoria	86	18.2%
Western Australia	51	10.8%
***Survey mode:***		
Online	205	43.3%
Telephone	268	56.7%
***Intervention:***		
Healthy Eating	241	51.0%
Physical Activity	232	49.0%
***Mean number of educators by employment status***		***Mean no. per service (SD):***
Full-time	473	9 (8)
Part-time^b^	472	7 (8)
Casual^b^	469	3 (4)
***Mean no. of children in service***	473	59 (31)

*SD* Standard Deviation

^a^Other positions include: Service owner, Room Leader, and Educator

^b^Missing responses for these characteristics

### Barriers and facilitators according to the IMPRESS-C scale domains

Each of the 473 services responded to one of the 13 physical activity and healthy eating interventions, depending on the intervention they were currently implementing. The physical activity interventions included; activities with children (*n* = 70), Energisers (*n* = 97), fundamental movement skills (*n* = 30), and outdoor time with a planned activity (*n* = 35). The healthy eating interventions included; two or more serves of fruit per day (*n* = 11), two or more serves of vegetables per day (*n* = 48), exposure to different vegetables (*n* = 16), healthy eating-themed special days (*n* = 13), interactive healthy eating activities (*n* = 53), observations of children’s lunchboxes (*n* = 9), planned healthy eating lessons (*n* = 17), play based healthy eating activities (*n* = 50), and strategies to encourage consumption of age appropriate beverages (*n* = 24).

The IMPRESS-C scale resulted in the identification of a number of potential barriers and facilitators including; (1) Outer Contextual Factors domain, which comprised of the sociopolitical context, funding environment and availability, external partnerships and leadership and the extent to which the intervention fits with national, state or local priorities, needs and values; (2) Inner Contextual Factors domain, which involves programme champions, organisational leadership/support, organisational readiness/resources, and organisational stability such as staffing attrition; (3) Processes domain which comprises of partnership/engagement, training/supervision/support and programme evaluation/data, adaption and communications and strategic planning; (4) Characteristics of the Intervention domain, which focuses on the adaptability/fidelity of the intervention, its fits within the context/population/organisation, the perceived benefits of the intervention and the perceived need for the intervention [41]. The Outer Contextual Factors and Processes domains had the lowest mean scores of less than four on average, indicating they may be considered barriers to sustainability (see Table 3). Inner Contextual Factors and the Characteristics of the Interventions, had the highest mean score of more than four on average, indicating they may be considered facilitators to sustainability (see Table 3).

**Table 3:** Descriptive statistics of the IMPRESS-C domains and identification of barriers and facilitators to sustainability

IMPRESS-C Domain	Lower quartile	Upper quartile	Min	Max	Mean (SD)
Outer contextual factors	3.67	4.33	1.00	5.00	3.93 (0.63)
Inner contextual factors	3.88	4.50	1.00	5.00	4.09 (0.52)
Processes	3.40	4.00	1.00	5.00	3.78 (0.64)
Characteristics of the intervention	4.00	4.40	2.70	5.00	4.16 (0.46)

*SD* Standard Deviation

When looking at the individual interventions, the number of responses ranged from nine to 98. As shown in Table 4, for physical activity, the scores within the ‘Energisers’ intervention had the lowest scores (means ranging from 3.61 to 4.16), while ‘activities with children’ had higher scores across all domains (means ranging from 3.98 to 4.28). For the IMPRESS-C domains, across physical activity interventions, the Outer Contextual Factors (means ranging from 3.79 to 4.02) and Processes (means ranging from 3.61 to 3.98) domains means were consistently lower than the Inner Contextual Factors (means ranging from 4.00 to 4.20) and Characteristics of the Intervention (means ranging from 4.16 to 4.30) domain means and (see Table 4).

**Table 4:** Descriptive statistics for services delivering physical activity interventions

	**IMPRESS-C Domain**																			
	**Outer contextual factors**					**Inner contextual factors**					**Processes**					**Characteristics of the intervention**				
**Physical activity interventions**	***n***	**Mean (SD)**	**IQR**	**Min**	**Max**	***n***	**Mean (SD)**	**IQR**	**Min**	**Max**	***n***	**Mean (SD)**	**IQR**	**Min**	**Max**	***n***	**Mean (SD)**	**IQR**	**Min**	**Max**
*Activities with children*	72	4.02 (0.57)	4.00	2.67	5.00	72	4.20 (0.44)	4.00	2.88	5.00	72	3.98 (0.59)	4.00	2.40	5.00	72	4.28 (0.44)	4.10	3.10	5.00
*Energisers*	98	3.79 (0.60)	4.00	2.33	5.00	98	4.00 (0.46)	4.00	2.62	5.00	98	3.61 (0.70)	3.80	1.20	5.00	98	4.16 (0.34)	4.00	3.60	5.00
*Fundamental movement skills*	30	3.96 (0.71)	4.00	2.33	5.00	30	4.15 (0.56)	4.12	3.12	5.00	30	3.82 (0.73)	4.00	2.20	5.00	30	4.30 (0.44)	4.10	3.60	5.00
*Outdoor time with a planned activity*	35	3.93 (0.82)	4.00	1.00	5.00	34	4.07 (0.78)	4.00	1.00	5.00	34	3.81 (0.57)	4.00	2.40	5.00	34	4.21 (0.44)	4.00	2.90	5.00

*SD* Standard Deviation, *IQR* Inter Quartile Range, *Min* Minimum, *Max* Maximum

While in Table 5, for healthy eating, scores were the lowest across all domains for ‘strategies to encourage consumption of age-appropriate beverages’ (mean scores ranging between 3.42 and 3.94) and generally, the highest scores were found in the ‘healthy eating-themed special days’ intervention (means ranging from 4.13 to 4.38). For the IMPRESS-C domains, across healthy eating interventions, the Outer Contextual Factors (means ranging from 3.62 to 4.21) and Processes (means ranging from 3.42 to 4.14) domains also had lower means compared to the Inner Contextual Factors (means ranging from 3.85 to 4.29) and Characteristics of the Intervention (means ranging from 3.94 to 4.38) domains (see Table 5). In general, healthy eating interventions had higher means in all domains compared to physical activity interventions.

**Table 5:** Descriptive statistics for services delivering healthy eating interventions

	**IMPRESS-C Domain**																			
	**Outer contextual factors**					**Inner contextual factors**					**Processes**					**Characteristics of the intervention**				
**Healthy eating interventions**	***n***	**Mean (SD)**	**IQR**	**Min**	**Max**	***n***	**Mean (SD)**	**IQR**	**Min**	**Max**	***n***	**Mean (SD)**	**IQR**	**Min**	**Max**	***n***	**Mean (SD)**	**IQR**	**Min**	**Max**
*Two or more serves of fruit per day*	12	4.21 (0.36)	0.33	3.67	5.00	12	4.15 (0.43)	0.63	3.50	4.88	12	3.80 (0.44)	0.55	2.80	4.40	12	4.22 (0.34)	0.45	3.90	4.90
*Two or more serves of vegetables per day*	49	3.99 (0.43)	0.67	3.00	5.00	49	4.10 (0.43)	0.47	3.00	5.00	48	3.95 (0.51)	0.60	3.00	5.00	48	4.16 (0.38)	0.38	3.10	5.00
*Exposure to different vegetables*	15	3.94 (0.60)	0.67	2.67	4.67	16	3.94 (0.43)	0.38	3.00	4.88	16	3.52 (0.55)	1.00	2.60	4.40	16	4.08 (0.49)	0.40	3.00	5.00
*Healthy eating-themed special days*	13	4.13 (0.59)	1.00	3.33	5.00	13	4.20 (0.59)	0.88	3.00	5.00	13	4.14 (0.54)	0.80	3.00	5.00	13	4.38 (0.37)	0.65	4.00	5.00
*Interactive healthy eating activities*	53	3.98 (0.70)	0.67	2.00	5.00	53	4.02 (0.47)	0.31	3.12	5.00	53	3.71 (0.74)	0.60	1.00	5.00	53	4.06 (0.41)	0.15	3.00	5.00
*Observations of children’s lunchboxes*	9	3.67 (0.58)	1.00	3.00	4.67	9	4.12 (0.57)	0.69	3.00	5.00	9	3.53 (0.69)	2.40	2.40	4.80	9	4.27 (0.66)	1.00	3.00	5.00
*Planned healthy eating lessons*	18	3.84 (0.68)	0.92	2.33	5.00	18	4.14 (0.57)	0.75	2.62	5.00	18	3.64 (0.68)	0.80	2.60	5.00	18	4.09 (0.39)	0.25	3.30	5.00
*Play based healthy eating activities*	51	4.03 (0.72)	1.00	2.00	5.00	51	4.29 (0.52)	1.75	3.25	5.00	51	3.96 (0.62)	0.80	2.60	5.00	51	4.32 (0.50)	0.80	3.30	5.00
*Strategies to encourage consumption of age-appropriate beverages*	24	3.62 (0.54)	0.67	2.33	4.33	24	3.85 (0.64)	0.75	2.75	5.00	24	3.42 (0.62)	1.00	2.00	4.40	23	3.94 (0.54)	0.30	2.70	5.00

*SD* Standard Deviation, *IQR* Inter Quartile Range, *Min* Minimum, *Max* Maximum

### Association between barriers/facilitators of sustainability and ECEC service characteristics

Linear regression analyses revealed no statistically significant independent associations between the overall means scores of IMPRESS-C domains and service characteristics including socio-economic status, Accessibility/Remoteness Index of Australia, type of service, service size, days of operation and service operating hours and time delivering its health program for at least two years (see Table 6). The odds of a service having delivered its health program for at least two years was 52% lower for each additional score of the “Characteristics of the Intervention” domain.

**Table 6:** Linear regression estimates of the association between the service characteristics and barriers and facilitators to the sustainability of physical activity and healthy eating interventions

**Factor**	Domain	Effect size (OR^1^ or mean difference^2^)	95% CI	*p*-value
Socio-economic status (reference = higher socio-economic status)	Outer Contextual Factors	0.96	0.72, 1.28	0.767
	Inner Contextual Factors	0.94	0.66, 1.34	0.738
	Processes	1.06	0.80, 1.40	0.696
	Characteristics of the Intervention	0.96	0.63, 1.47	0.867
Accessibility/Remoteness Index of Australia^1^ (reference = remote)	Outer Contextual Factors	1.10	0.62, 1.94	0.741
	Inner Contextual Factors	0.91	0.45, 1.85	0.803
	Processes	1.51	0.90, 2.54	0.122
	Characteristics of the Intervention	0.98	0.42, 2.28	0.967
Type of service^1^ (reference = preschool)	Outer Contextual Factors	1.18	0.73, 1.91	0.511
	Inner Contextual Factors	0.95	0.52, 1.73	0.862
	Processes	1.02	0.63, 1.64	0.950
	Characteristics of the Intervention	0.73	0.36, 1.50	0.393
Size^1^ (reference = < = 80 children)	Outer Contextual Factors	1.24	0.85, 1.80	0.262
	Inner Contextual Factors	1.14	0.73, 1.77	0.574
	Processes	1.31	0.91, 1.89	0.145
	Characteristics of the Intervention	0.91	0.53, 1.54	0.716
Days of operation^2^	Outer Contextual Factors	0.00	-0.02, 0.03	0.795
	Inner Contextual Factors	-0.01	-0.04, 0.02	0.632
	Processes	0.00	-0.02, 0.03	0.678
	Characteristics of the Intervention	-0.03	-0.06, 0.01	0.139
Service opening hours^2^	Outer Contextual Factors	-0.04	-0.24, 0.15	0.653
	Inner Contextual Factors	-0.14	-0.37, 0.10	0.250
	Processes	0.07	-0.12, 0.26	0.461
	Characteristics of the Intervention	-0.20	-0.49, 0.08	0.161
Delivered program (Reference: < 2 years)	Outer Contextual Factors	1.19	0.76, 1.88	0.445
	Inner Contextual Factors	0.93	0.52, 1.66	0.817
	Processes	0.89	0.56, 1.41	0.616
	Characteristics of the Intervention	0.48	0.24, 0.95	0.035

*OR* Odds Ratio, *CI* Confidence Interval

Reference groups: Socio-economic status – higher socio-economic status, Accessibility/Remoteness Index of Australia – remote, service type—preschool, size—<  = 80 children, Delivered program—< 2 years, statistical significance =  ≤ 0.05

^1^Effect size calculated using OR

^2^Effect size calculated using mean difference

## Discussion

This study describes the barriers and facilitators to the sustainability of physical activity and healthy eating interventions for children in ECEC settings in the Australian context. It uses a validated measure of sustainability determinants completed by service executives and explored the association between intervention sustainability factors and ECEC service characteristics.

Overall, amongst the 473 ECEC services, our analysis found that the Outer Contextual Factors and Processes domains scored the lowest suggesting that external support such as existing policies and regulations, the funding environment and service partnerships/engagement, training and adaptation processes that may potentially address these barriers are essential for overall intervention sustainability. Our analysis found that Inner Contextual Factors and Characteristics of the Intervention domains scored the highest, suggesting that internal support such as financial resources, program champions, and organisational support, as well as the interventions adaptability, and fit within the context and population may be important for these interventions to be sustained. Such findings are perhaps unsurprising given that Outer Contextual factors (inadequate existing policies or guidelines, limited future external financial support), and Processes (insufficient professional development opportunities, and limited training or stakeholder engagement) have been previously reported as potential barriers to intervention sustainability [35, 44, 59–63]. Moreover, our results align with previous review findings highlighting the critical contribution of human and financial resources, as well as ongoing professional development to the sustainability of obesity prevention interventions more broadly [64]. Similarly, in the school setting, insufficient funding, equipment, materials and/or physical space, as well as a lack of training have been identified as barriers to intervention sustainability [34, 39, 44].

To address the Outer Contextual Factors domain, future interventions may need to consider alignment with existing outer context levers. This includes ensuring alignment with policies, guidelines, or standards and working with accreditation agencies to embed such programs within National Quality Standards. Previous research with ECECs suggests that alignment with such policies and guidelines are important predictors of implementation and ongoing delivery of programs within usual context [65–67]. Unsurprisingly outer contextual factors including funding and external policies also emerged as key predictors of sustainment consistent with previous literature [35, 44, 59–63]. Partnerships with external funders or supporters of the intervention, including universities, health, government and non-government organisations may be one way to address such gaps in funding to support ongoing implementation and sustainment [60]. Importantly, such funding should focus on capacity building and enhancing sustainment specifically by ensuring procedures are bedded into organisational policies, and that capacity building and training efforts are accessed in an ongoing way to address staff turnover [60]. Further, it has been suggested that these efforts should commence during the inception of the intervention focusing on ensuring a congruence between the intervention and its context, rather than addressing sustainability at the end of the program [38, 60, 68, 69].

Additionally, the regression analyses, which controlled for various ECEC service characteristics found no statistically significant association between socioeconomic status, remoteness, type of service, service size, days of operation and service operating hours. This suggests that barriers to sustainability may not be contingent upon any specific service characteristic. These findings were surprising given that prior studies have recommended tailoring interventions to service characteristics, although our analysis via linear regression may not have identified a meaningful difference and linear regression to determine association is recommended [44, 70, 71]. However, given that the majority of our sample were long-day care services (91%) located in in urban areas (93%) it is possible that further research with a broader sample of ECEC services located in rural/regional settings are needed. Our results found a statistically significant association between the number of years services deliver their interventions and the Characteristics of the Intervention domain (*p* = 0.035), suggesting that this domain could be an important facilitator to length of implementation. Other research suggests that an intervention which lends itself to being adaptable to the service, meets a perceived need, is low-intensive and low-cost as to not burden the service, is more likely to be delivered ≥ 2 years [28].

### Strengths and limitations

This study describes for the first time, factors associated with sustainability of a range of physical activity and healthy eating interventions delivered in ECEC services in the Australian context using the validated IMPRESS-C. It further reinforces the domains related to Outer Contextual Factors and Processes as needing to be considered as a way of ensuring sustainment of these interventions.

A strength of this study is the use of a validated measure to assess determinants influencing sustainability of physical activity and healthy eating interventions in ECEC settings from the perspective of the service executive. However, a number of limitations exist. Firstly, while the study included an appropriate sample size of ECEC services, there were limited numbers of services within each specific intervention (ranging from nine to 98). Additionally, the majority of services were located in New South Wales (NSW) (42.0%), which may affect the generalisability of the findings to other states. Similarly, as majority of our sample were long-day care services in urban areas, future studies should employ more representative sampling procedures to ensure the findings are generalisable to all Australian ECECs. Secondly, many of the responses were primarily on the upper end of the scale, indicating a possible ceiling effect and limited range of responses captured. Thirdly, the reliance on quantitative measurement of determinants for the intervention implementation may have resulted in reporting or recall bias and may not be reflective of actual practice. In future, a mixed-methods study assessing these determinants may be used to help mitigate information bias [72]. Lastly, the study assessed the sustainability determinants from the perspective of nominated supervisors. To obtain a full range of perceptions and a comprehensive understanding of factors influencing intervention sustainability, it is important to also capture determinants from the implementer perspective of service educators. Educators possess an understanding of the day-to-day operations and delivery of interventions and what may impact their ongoing delivery [73, 74]. Nevertheless, the data generated by this study provides valuable insights into the primary barriers and facilitators experienced by ECEC services to inform future efforts to improve sustainability of such interventions.

### Implications for research, policy and practice

This study found that key factors from the Outer Contextual Factors and Processes domains including external funding, guidelines, community partnerships, and adequate training, may be important for the sustainability of ECEC-based physical activity and healthy eating interventions. While such findings are perhaps unsurprising, the growing body of research on sustainability determinant and strategies is starting to offer guidance for program developers and implementers on how to address these potential barriers. Nathan and colleagues (2022) have suggested a number of amendments to implementation strategies that could be of use to inform the development of future strategies targeting the factors identified here [75].

The authors have undertaken a rigorous systematic mapping process to develop sustainment strategies to primarily address the Outer Contextual factors and Processes domains [76]. This pilot randomised controlled trial utilised strategies including but not limited to identifying opinion leaders, engaging with family members, providing educational materials on external policies and guidelines, and embedding change into policy, which will likely provide initial insight into the potential impact of sustainment strategies on addressing such determinants.

## Conclusions

For population health impact to be realised, interventions in ECEC settings must be effectively implemented and sustained. It is recommended that future physical activity and healthy eating interventions in these settings consider Outer Contextual Factors and Processes domains, and their deliberate integration into intervention design. Recommendations to address these barriers include ensuring services are equipped with sufficient information of state requirements and funding opportunities, as well as the potential to implement the intervention into sector policy to ensure continuous training and improvement of ECEC service staff. Our findings hold significant relevance for policy makers, interventionists, researchers, and health promotion staff involved in the design of physical activity and healthy eating interventions in ECEC settings.

## Data Availability

The datasets used and/or analysed during the current study are available from the corresponding author on reasonable request.

## Appendix 1

**Table 7:** Healthy eating interventions included and their criteria

Name of intervention	Criteria
Planned healthy eating lessons	Service provides planned healthy eating education sessions at least monthly
Observation of children’s lunchbox	Service observes children’s lunchboxes to ensure that they are consistent with the Australian Dietary Guidelines at least 1 to 2 times per week • Service provides feedback to families when lunchboxes are not consistent at least weekly
2 or more serves of vegetables per day	Service provides at least 2 services of vegetables to each child per day
Interactive healthy eating activities	Service delivers an interactive healthy eating activity at least once per month
Exposure to different vegetables	Service is exposing children to different vegetables as part of experiential learning at least once per month
Play based healthy eating activities	Service delivers play based health eating activities at least once per month
Healthy eating-themed special days	Service provides a healthy eating themed special day at least once per month
2 or more service of fruit per day	Service provides at least 2 serves of fruit to each child per day
Strategies to encourage consumption of age-appropriate beverages	Service implements strategies to encourage children to consumer age-appropriate beverages at least 2 times per week • Service provides water to children, and may provide reduced fat milk to children aged 2–6 years • Service does not provide sugar-sweetened beverage to children (e.g. fruit juice, cordial, flavoured milk or soft drink) • Service implements strategies to encourage children to consume age-appropriate beverages including water and milk every day • Educators role model healthy drink choices • Drinks provided/allowed by the service are consistent with the Australian Dietary Guidelines or Caring for Children Guidelines

**Table 8:** Physical activity interventions included and their criteria

Name of intervention	Criteria
Energisers	Service delivers an educator led energiser in the room on more than one day per week
Activities with children	Service engages children with activities at least once per week
Fundamental movement skills	Service provides an activity designed to intentionally teach and develop the various fundamental movement skills on at least one day per week
Outdoor time with a planned activity	Service delivers a planned outdoor time with a specific activity at least once per week

## Appendix 2

**Table 9:** ECEC CATI executive scale sustainability items – IMPRESS-C [41]

**Outer contextual factors**	**Completely Disagree**	**Disagree**	**Neither agree nor disagree**	**Agree**	**Completely agree**
1. My service governing body has a policy or guideline regarding the ongoing delivery of “the program” that my service follows. (Note: A governing body refers to an educational department or authority e.g., Australian Children's Education & Care Quality Authority).	1	2	3	4	5
2. My service has external partnerships that provide support for the ongoing delivery of “the program” within my service (Note: Examples of partnerships include national authorities, government agencies, councils and health organisations).	1	2	3	4	5
3. “The program” aligns with the priorities of my wider service community. (Note: service community refers to administrators, teachers/educators, staff members, children, their parents/guardians and families directly involved with your service).	1	2	3	4	5
**Inner contextual factors**	**Completely Disagree**	**Disagree**	**Neither agree nor disagree**	**Agree**	**Completely agree**
4. There are program champions in my service who positively influence others to continue to deliver “the program”. (Note: a champion is a peer representative that drives the continued delivery of the program within the service.	1	2	3	4	5
5. Management at my service support the ongoing delivery of “the program”.	1	2	3	4	5
6. Management at my service support the training of educators to enable the ongoing delivery of “the program”.	1	2	3	4	5
7. My service allocates sufficient space to support the ongoing delivery of “the program”.	1	2	3	4	5
8. My service has sufficient equipment to support the ongoing delivery of “the program”.	1	2	3	4	5
9. My service has sufficient funding to support the ongoing delivery of “the program”.	1	2	3	4	5
10. My service allocates sufficient time to support the ongoing delivery of “the program”.	1	2	3	4	5
11. My service would be able to continue to deliver “the program” if there was a change of leaders (e.g., management or champions) at our service.	1	2	3	4	5
**Processes**	**Completely Disagree**	**Disagree**	**Neither agree nor disagree**	**Agree**	**Completely agree**
12. Educators at my service receive sufficient formal training to support the ongoing delivery of “the program”.	1	2	3	4	5
13. My service is involved with collecting information and providing feedback to educators regarding my service’s performance in “the program”. Note: This may be collected in the form of teacher/educator or child surveys, or room observations	1	2	3	4	5
14. My service has a process to evaluate how well “the program” aligns with our priority areas and if it does not fit, it adapts “the program” as needed.	1	2	3	4	5
15. My service has a documented plan to continue the delivery of “the program” long-term.	1	2	3	4	5
16. My service promotes the ongoing delivery of “the program” to the wider service community e.g., through a website or newsletter. (Note: service community refers to administrators, teachers/educators, staff members, children, their parents/guardians and families directly involved with your service).	1	2	3	4	5
**Characteristics of the intervention**	**Completely Disagree**	**Disagree**	**Neither agree nor disagree**	**Agree**	**Completely agree**
17. My service is able to adapt “the program” if resources/equipment are reduced.	1	2	3	4	5
18. My service is able to adapt “the program” to suit the service environment.	1	2	3	4	5
19. I can easily adapt “the program” to fit within my normal schedule.	1	2	3	4	5
20. “The program” is appropriate for my service, regardless of the socio-demographic region my service resides in.	1	2	3	4	5
21. “The program” is culturally appropriate for children at my service.	1	2	3	4	5
22. “The program” is widely accepted within my service by educators.	1	2	3	4	5
23. “The program” is easily delivered within my service.	1	2	3	4	5
24. I believe “the program” helps to improve the health of children at my service.	1	2	3	4	5
25. The cost to deliver “the program” in my service is acceptable.	1	2	3	4	5
26. Delivering “the program” is as important as other learning outcomes specified within the Early Years Learning Framework e.g., encouraging children to be confident and involved learners.	1	2	3	4	5

## Abbreviations

ACECQA: Australian Children Education and Care Quality Authority
EBI: Evidence-Based Interventions
ECEC: Early Childhood Education and Care
HNELHD: Hunter New England Local Health District
HREC: Human Research Ethics Committee
IMPRESS-C: Integrated Measure of PRogram Element SuStainability in Childcare Settings
IQR: Interquartile Range
LCL: Lower Control Limit
NSW: New South Wales
OR: Odds Ratio
SD: Standard Deviation
SEIFA: Socio-Economic Indexes for Areas
UCL: Upper Control Limit
WHO: World Health Organization
